# Genomic Features of *Rickettsia heilongjiangensis* Revealed by Intraspecies Comparison and Detailed Comparison With *Rickettsia japonica*

**DOI:** 10.3389/fmicb.2019.02787

**Published:** 2019-12-06

**Authors:** Kentaro Kasama, Hiromi Fujita, Seigo Yamamoto, Tadasuke Ooka, Yasuhiro Gotoh, Yoshitoshi Ogura, Shuji Ando, Tetsuya Hayashi

**Affiliations:** ^1^Department of Bacteriology, Faculty of Medical Sciences, Kyushu University, Fukuoka, Japan; ^2^Mahara Institute of Medical Acarology, Tokushima, Japan; ^3^Frontier Science Research Center, University of Miyazaki, Miyazaki, Japan; ^4^Department of Microbiology, Graduate School of Medical and Dental Sciences, Kagoshima University, Kagoshima, Japan; ^5^Department of Virology-I, National Institute of Infectious Diseases, Tokyo, Japan

**Keywords:** *Rickettsia heilongjiangensis*, *Rickettsia japonica*, spotted fever group rickettsia, genome sequence, intraspecies genomic diversity

## Abstract

*Rickettsia heilongjiangensis* is the causative agent of Far-Eastern spotted fever (FESF). In Japan, a human case of FESF was identified in Sendai in Miyagi Prefecture in 2008, and *R. heilongjiangensis* bacteria were isolated from *Haemaphysalis concinna* ticks collected in the suspected geographical area of infection. Although the intraspecies genome diversity of Rickettsia has been poorly investigated, our recent analysis revealed extremely low genomic diversity of *R. japonica*, the agent of Japanese spotted fever, which is a close relative of *R. heilongjiangensis*. In this study, to investigate the genomic diversity of *R. heilongjiangensis* and understand the genetic relationship between Japanese and Chinese isolates, we sequenced three isolates from *H. concinna* ticks collected in Sendai and one isolate from a *H. concinna* tick collected in Inner Mongolia, China, and performed genomic comparisons between these isolates and strain 054, the type strain isolated from a *Dermacentor silvarum* tick in Heilongjiang Province, China. Although the three Japanese strains were isolated in 2008, 2009, and 2012, their genome sequences were identical, indicating that *H. concinna* ticks carrying a single *R. heilongjiangensis* clone have been distributed in Sendai, Japan. Among the five *R. heilongjiangensis* isolates, only 81 SNPs and 13 insertion/deletion sites were identified, despite the significant differences in these isolates both geographically and temporally. A significant portion of the 81 SNPs (16/81) were found to be recombinogenic. These results indicate low genomic diversity of *R. heilongjiangensis*, as observed in *R. japonica*. We further performed a detailed genomic comparison of *R. heilongjiangensis* and *R. japonica* to accurately define conserved and species-specific genes. This analysis revealed that although notable variations were found in the genomic loci encoding RelA/SpoT family proteins and tandem repeats in major surface proteins, there was only a small difference in the gene repertoire between the two species, suggesting that SNPs and small InDels are responsible for the functional or physiological differences between the two species, if present. Through this analysis, several species-specific genomic regions that can serve as ideal PCR targets for distinguishing *R. heilongjiangensis* and *R. japonica* were also identified.

## Introduction

Rickettsia are Gram-negative, obligate intracellular bacteria, some of which are known agents of arthropod-borne infectious diseases. These bacteria are divided into the spotted fever group (SFG), the typhus group (TG), and the ancestral group (AG) according to their phylogeny (Parola et al., 2013; Diop et al., 2019). The AG Rickettsia are sometimes divided into two subgroups, the *R. belli* and *R. canadensis* groups. SFG Rickettsia include more than 25 validated species, with additional new species or subspecies being proposed (Parola et al., 2013; Diop et al., 2019). The designation of a “transitional group” has also been proposed for a sublineage of SFG that includes *R. felis*, *R. akari*, and their close relatives (Gillespie et al., 2007).

Among SFG Rickettsia, at least 24 species/subspecies are known or suspected to be pathogenic to humans and cause tick-borne diseases referred to as “spotted fevers” (Parola et al., 2013). The life cycles of SFG Rickettsia are closely associated with ticks. Ticks act as both vectors and reservoirs for most SFG Rickettsia; the bacteria are maintained in ticks through transstadial and transovarial transmission (Socolovschi et al., 2009). Importantly, most SFG members are thought to have adapted to a specific tick species (Parola et al., 2013). Therefore, the distribution of most species is restricted to a relatively limited geographic region, where specific tick species are distributed, and each causes a region-specific “spotted fever.”

*Rickettsia heilongjiangensis* is the causative agent of Far-Eastern spotted fever (FESF) for humans. The endemic areas of FESF have been considered to be northeastern China (Liu et al., 2016), Siberia, and far-eastern Russia (Igolkina et al., 2018). Strain 054, the type strain of this species, was first isolated in Heilongjiang Province, China, in 1982 (Zhang et al., 2000). Determination of its complete genome sequence and subsequent phylogenetic analysis revealed that *R. heilongjiangensis* is closely related to *R. japonica*, the agent of Japanese spotted fever (JSF) (Duan et al., 2011). In Japan, a human case of FESF was identified in Miyagi Prefecture in 2008 (Ando et al., 2010). The diagnosis of this patient was performed by PCR and sequencing analysis of the *rompA*, *gltA*, and 17-kDa antigen genes because bacterial isolation was not succeeded. However, *R. heilongjiangensis* was isolated from a *Haemaphysalis concinna* tick collected in a suspected geographic area of infection (Ando et al., 2010). Multiple *R. heilongjiangensis* isolates were further obtained from *H. concinna* ticks collected in this area from 2008 to 2012 (Fujita, unpublished data), suggesting that *H. concinna* ticks carrying *R. heilongjiangensis* inhabit this region.

In Japan, JSF is the main rickettsiosis; thus, it is important to discriminate *R. japonica* and *R. heilongjiangensis*. However, the two species are very closely related and no PCR or serological tests suitable for this purpose have been developed. This is because, while the genetic diversity of *R. japonica* has been recently analyzed using high-quality whole-genome sequences (WGSs) of 31 *R. japonica* isolates obtained in various regions of Japan (Akter et al., 2017) that of *R. heilongjiangensis* has not yet been investigated.

Here, we sequenced three Japanese and one Chinese *R. heilongjiangensis* isolate and performed a WGS-based high-resolution phylogenetic analysis including strain 054 to understand the genomic diversity of this species and the genetic relationships of Japanese and Chinese isolates. A detailed genomic comparison of *R. heilongjiangensis* and *R. japonica* was also performed to better understand the genomic difference between the two species and to identify genetic traits that can be used to discriminate the two species.

## Materials and Methods

### Bacterial Isolates

**Figure 1:**
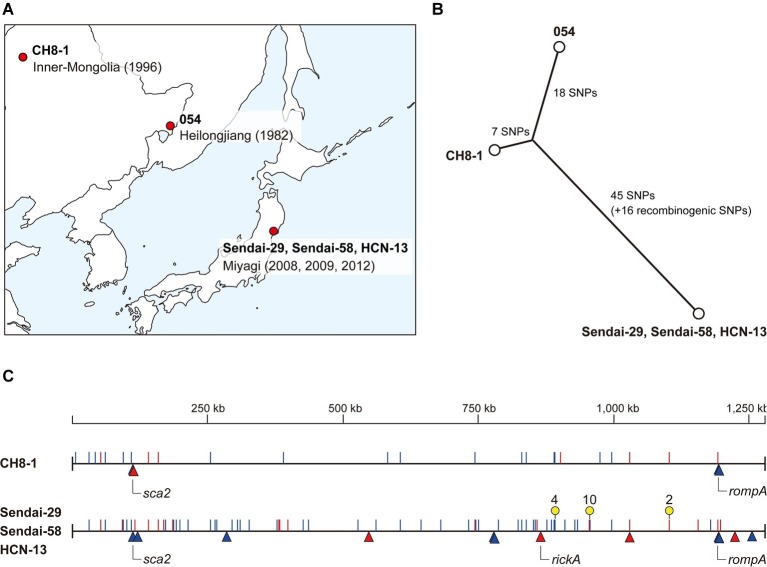
Intraspecies genomic comparison of *R. heilongjiangensis.* **(A)** Geographic information of the *R. heilongjiangensis* isolates analyzed in this study. **(B)** Phylogenetic relationships and SNP distances of the five *R. heilongjiangensis* isolates. **(C)** Genomic locations of all SNPs and InDels identified in the Sendai strain and CH8-1. Strain 054 (the top line) was used as the reference. SNPs and InDel are indicated by lines (blue, transition; red, transversion) and triangles (blue, insertion; red, deletion), respectively. Regions marked by yellow pins correspond to the SNP clusters (two or more SNPs within 3- to 25-bp distances) that were probably introduced by recombination.

The *R. heilongjiangensis* isolates analyzed in this study are listed in Table 1. We sequenced four *R. heilongjiangensis* isolates: one (CH8-1) isolated from a *H. concinna* tick collected in the foothills of the Greater Khingan Range, Inner Mongolia, China, in 1996 (Fujita et al., 2002; Ando et al., 2010), and three (Sendai-29, Sendai-58, and HCN-13) isolated from *H. concinna* ticks collected in Sendai City, Miyagi, Japan, in 2008, 2009, and 2012, respectively (Table 1; Figure 1A). The sites of tick collection for the Japanese isolates were within 2.5 km of each other. CH8-1 and Sendai-29 were isolated in our previous study (Ando et al., 2010), and Sendai-58 and HCN-13 were isolated in this study by the same procedure. Strain 054, the type strain of *R. heilongjiangensis*, was isolated from a *Dermacentor silvarum* tick collected in Heilongjiang Province, China (Zhang et al., 2000), and was previously sequenced (Duan et al., 2011, 2014). The genome sequence of strain 054 (accession no. NC_015866.1) was downloaded from the National Center for Biotechnology Information (NCBI) RefSeq database^1^.

**Table 1:** The *R. heilongjiangensis* strains analyzed in this study.

Strain/isolate name	Date of isolation (y/m/d)	Location of tick collection	Host tick		Genome information				Reference
			Species	Stage/Sex	Genome size (bp)	CDS (pseudogene)	rRNA/tRNA genes	Accession No.	
054	1982	China, Heilongjiang	*D. silvarum*	No information	1,278,471	1,485 (275)	3/33	NC_015866.1	Zhang et al. (2000); Duan et al. (2011)
CH8-1	1996/5/27	China, Inner Mongolia	*H. concinna*	Adult, male	1,279,380	1,485 (276)	3/33	AP019862	Fujita et al. (2002)
Sendai-29	2008/9/23	Japan, Miyagi	*H. concinna*	Nymph	1,279,369	1,485 (276)	3/33	AP019864	Ando et al. (2010)
Sendai-58	2009/3/29	Japan, Miyagi	*H. concinna*	Adult, male	1,279,369	1,485 (276)	3/33	AP019865	This study
HCN-13	2012/6/23	Japan, Miyagi	*H. concinna*	Adult, female	1,279,369	1,485 (276)	3/33	AP019863	This study

### Genomic DNA Preparation

The bacterial cells were inoculated to L929 cells and incubated in Eagle’s minimal essential medium (Nissui, Japan) containing 1% fetal bovine serum and 2 mM L-glutamine at 34°C for 7–10 days as previously described (Fujita, 2008). After centrifugation at 800 rpm for 5 min to remove L929 cell debris, the supernatants were centrifuged at 14,000 rpm for 10 min to recover bacterial cells. The obtained pellets were suspended in phosphate-buffered saline (PBS, pH 7.2). The cell suspensions were treated with DNase I (Takara Bio, Japan; 2,000 U/ml at a final concentration) for 1 h at 37°C to digest cell-free DNA. DNase I was inactivated by incubation at 65°C for 5 min, and EDTA (Nacalai Tesque, Japan) was added to the solutions at a final concentration of 2 mM. Bacterial genomic DNA was extracted using the DNeasy Blood and Tissue Kit (Qiagen, Japan) according to the manufacturer’s instructions.

### Genome Sequencing, Assembly, and Annotation

Sendai-29 was sequenced using Roche 454 GS-FLX Titanium to generate 354,422 single-end reads (average read length: 316 bp). A total of 64,428 paired-end (PE) reads were also generated from a mate-pair library (insert size: 8 kb). The read sequences were assembled with GS Assembler ver. 2.3 to obtain seven scaffolds. Among these scaffolds, six were found to be derived from mouse DNA by BLASTn analysis. Nine gaps in the remaining scaffold were filled by sequencing PCR products that spanned each gap using an ABI 3730 capillary sequencer to obtain a nearly complete sequence, which contained a single gap in a tandem repeat-containing region of the *rompA* gene. The genome was also sequenced using the Illumina MiSeq platform (PE reads; 150 bp ×2) to verify and correct the sequence.

Genomic DNA libraries of CH8-1, Sendai-58, and HCN-13 were prepared using the QIAseq FX DNA Library Kit (Qiagen) and sequenced on the Illumina MiSeq platform to obtain PE sequences (300 bp ×2). After adapter and low-quality sequences were trimmed using Platanus_trim ver. 1.07^2^, the reads were mapped to the mouse genome sequence (accession nos. NC_000067-NC000087, NC_005089) using Burrows-Wheeler-Aligner (BWA) ver. 0.7.15 (Li and Durbin, 2010) to filter mouse sequences. Unmapped reads were extracted using SAMtools ver. 1.3.1 (Li et al., 2009) and Picard tools^3^ and assembled using Platanus Assembler ver. 1.1.0 (Kajitani et al., 2014) to generate a single scaffold that contained two to six gaps in each genome. These gaps, except for that in the tandem repeat-containing region of the *rompA* gene, were closed by capillary sequencing of PCR products as described for Sendai-29. Sequence statuses of each genome, including total numbers of reads obtained, are summarized in Supplementary Table 1.

The sequences of the tandem repeat-containing regions in the *rompA* gene were determined using a MinION sequencer (Oxford Nanopore Technologies, UK). The 3.8-kb regions containing the tandem repeats were amplified with the primers RH_ompA_F (5′-aagccgctttattcaccacc-3′) and RH_ompA_R (5′-cgcacctagtgtgatcgtac-3′) using the PrimeSTAR GXL polymerase kit (Takara Bio Inc.). The PCR products were purified using the Wizard SV Gel and PCR Clean-Up system (Promega Corporation, USA). Sequencing libraries were prepared using the Rapid Barcoding Kit (Oxford Nanopore Technologies; SQK-RBK004) and sequenced using a MinION R9.4.1 flow cell. Sequence reads were base-called using Albacore ver. 2.3.1 and quality-filtered (score > 10, read length ≥ 2,000 bp) using Nanofilt ver. 2.1.1^4^. The filtered reads were assembled using Canu ver. 1.7 (Koren et al., 2017). The assembled sequences were verified by mapping the Illumina reads of each isolate using BWA and SAMtools.

The genome sequences determined in this study were annotated according to the RefSeq annotation for strain 054 (NC_015866.1). The complete genome sequences determined in this study have been deposited in DDBJ/EMBLE/NCBI under accession numbers AP019864 (Sendai-29), AP019865 (Sendai-58), AP019863 (HCN-13), and AP019862 (CH8-1). The raw Illumina reads have also been deposited in the DRA/SRA/ERA database under accession number DRA008226.

### Single Nucleotide Polymorphism and Insertion and Deletion Detection in the *R. heilongjiangensis* Genomes

Single nucleotide polymorphisms (SNPs) and insertion and deletions (InDels) were analyzed between 054, CH8-1, and Sendai-29 because the genomes of the three Japanese isolates were identical. The sequence of strain 054 was used as a reference for SNP and InDel calling, which was performed based on the alignments of the complete sequences constructed using NUCmer ver. 3.1 (Delcher et al., 2002) and MUMmer (Delcher et al., 1999), respectively. Annotation of SNPs was conducted using SnpEff ver. 4.2 (Cingolani et al., 2012).

### Genomic Comparison of *R. heilongjiangensis* and *R. japonica*

The genome sequences of *R. heilongjiangensis* Sendai-29 and *R. japonica* YH_M (Akter et al., 2017) were used for comparison. The average nucleotide identity according to the BLAST algorithm (ANIb) was calculated with JSpecies ver. 1.2.1 (Richter and Rossello-Mora, 2009). The numbers of SNPs and InDels were determined by constructing WGS alignments using NUCmer and MUMmer, respectively.

To compare the gene repertoires of the two strains using the dataset obtained from the same annotation platform, protein-coding sequences (CDSs) were identified using Prokka ver. 1.12 (Seemann, 2014). Ten and seven CDSs whose counterparts were apparently conserved but were not assigned by Prokka were manually assigned in *R. heilongjiangensis* and *R. japonica*, respectively. CDSs that were conserved in both species were identified by all-to-all blastp analysis (cutoff of >70% identity and > 30% length coverage). All identified CDSs are listed in Supplementary Table 2 with the “conserved/nonconserved” information for each CDS. In addition, we manually inspected the alignment of the two genomes to determine whether a CDS defined as “nonconserved” was truly specific to one strain or if a homologous nucleotide sequence was present in the other strain, but a corresponding CDS could not be assigned due to the presence of SNPs or small InDels. The functions of CDSs specific to one strain and those that were degraded in one strain (split into two or more CDSs or truncated) were predicted based on the results of homology searches using the NCBI blastp pipeline (see Supplementary Table 2 for the list of these CDSs and their predicted functions). The genome alignment of the two species was illustrated using GenomeMatcher ver. 2.205 (Ohtsubo et al., 2008).

### Analyses of Tandem Repeat Structures in the *rompA*, *sca1*, *sca2* Genes

We analyzed the inter- and intraspecies variation of tandem repeats in the *rompA*, *sca1*, and *sca2* genes of all available *R. heilongjiangensis* and *R. japonica* genomes. The genome sequences of *R. japonica* strains were downloaded from the NCBI RefSeq database (last accessed on 2019-01-07; accession numbers are listed in Supplementary Table 3). Tandem repeats in the three genes were identified using Tandem Repeat Finder (Benson, 1999).

## Results and Discussion

### General Genomic Features of the Four *R. heilongjiangensis* Isolates Sequenced in This Study

We determined the complete genome sequences of the three Japanese and one Chinese *R. heilongjiangensis* isolates. The three Japanese isolates (Sendai-29, Sendai-58, and HCN-13) were isolated from *H. concinna* ticks collected at very close locations (within 2.5 km) in Sendai city, Miyagi Prefecture. The Chinese isolate (CH8-1) was also isolated from a *H. concinna* tick collected in Inner Mongolia, China, in 1996 (Table 1; Figure 1A).

Although the three Japanese isolates were obtained in 2008, 2009, and 2012, their genome sequences were identical, showing no genomic variations. Therefore, they are hereafter collectively referred to as the “Sendai strain.” This result indicates that three isolates represent a single clone and that *H. concinna* ticks carrying this clone have been distributed in this region. This region is the suspected area, where the first Japanese FESF patient identified in 2008 was infected by *R. heilongjiangensis*. Although *R. heilongjiangensis* was not isolated from this patient, the patient was likely infected by this clone *via* a *H. concinna* tick bite. If so, there is a considerable risk of *R. heilongjiangensis* infections in this region. No FESF patients have been identified in other regions in Japan. *R. heilongjiangensis* carried by *H. concinna* ticks have not yet been isolated in other regions and no systematic investigation of the distribution of *H. concinna* ticks has been conducted. However, it is also possible that there are unidentified (hidden) FESF patients in Japan due to the poor understanding of spotted fever in the northern part of Japan, which is the nonendemic area of JSF. It will be important to systematically and continuously survey FESF patients and *R. heilongjiangensis*-carrying ticks in Japan.

The genome of the Sendai strain was comprised of a circular chromosome of 1,279,369 bp in length (GC content; 32.3%) and contained 1,485 CDSs (including 276 pseudogenes), three rRNA genes (5S, 16S, and 23S), and 33 tRNA genes. The genome size of CH8-1 was 1,279,380 bp, and the genome contained the same numbers of CDSs and rRNA and tRNA genes (Table 1). Plasmids were not found in either isolates, same as strain 054, which was isolated from a *D. silvarum* tick collected in Heilongjiang Province, China (Duan et al., 2011, 2014). Although it was previously reported that strain 054 contained many insertion sequences (ISs) (Duan et al., 2014), we identified no IS elements in the isolates sequenced in this study or in strain 054. Therefore, the *R. heilongjiangensis* genomes are devoid of IS elements. Prophages were also not detected in the *R. heilongjiangensis* genomes.

### Comparison of the Genomes of Japanese and Chinese *R. heilongjiangensis* Isolates

Comparison of the genomes of the Sendai strain and two Chinese isolates (CH8-1 and 054) revealed that the *R. heilongjiangensis* genome sequences were highly conserved, despite the fact that isolation conditions of the three isolates differed significantly geographically, temporally, and in terms of the host tick species. We identified only 86 SNP and 13 InDel sites at the WGS level (listed in Supplementary Tables 3, 4, respectively). The identified SNPs and InDels revealed the phylogenetic relationships and genetic distances between the three isolates. The Sendai strain differed from strain 054 by 79 SNPs and 12 InDels, while CH8-1 differed from strain 054 by 25 SNPs and four InDels (Figures 1B,C). However, the Sendai strain contained three genomic regions (nucleotide positions in strain 054: 888,963–888,979, 955,989–956,014, and 1,155,544–1,155,547) with multiple accumulated SNPs (two or more SNPs within 3- to 25-bp distances) (Figure 1C; Supplementary Table 4). In addition, many of the SNPs in these regions (10/16) were generated by transversion, sharply contrasting with the rate among other SNPs (18/70). These features indicate that the three SNP clusters are most likely to have been introduced by recombination. Therefore, the actual genetic distances between the Sendai strain and the two Chinese strains should be considered excluding these recombinogenic SNPs (Figure 1C).

Among the 13 InDel sites, three were shared by the Sendai strain and CH8-1, and 9 were found only in the Sendai strain (Figure 1C; Supplementary Table 5). Importantly, the three InDel sites shared by the two strains were associated with tandem repeats in three genes encoding surface proteins: *rickA*, *rompA*, and *sca2*. Thus, the presence of these InDels does not reliably indicate the phylogeny of the strains. One InDel site unique to CH8-1 was also associated with tandem repeats in the *sca2* gene.

The very low level of genomic diversity observed for *R. heilongjiangensis* is comparable to that observed in the analysis of 31 *R. japonica* isolates obtained in various regions in Japan: 32–39 SNPs between Lineage I (the major lineage) and Lineage II (a minor lineage) and 59–66 SNPs between Lineage I and Lineage III (another minor lineage) (Akter et al., 2017). Although the two Chinese isolates were isolated from different tick species, they are more closely related than the Chinese and Japanese isolates are, suggesting a stronger geographic association than the host tick-association. However, such evolutionary relationships have not yet been described for other rickettsia species and other bacteria with similar tick-associated life cycles (transovarial and transstadial transmission and horizontal transmission *via* blood feeding of infected animals), such as *Coxiella burnetii* and related species (Eldin et al., 2017) and *Borrelia miyamotoi* (Wagemakers et al., 2015). In addition, as *R. heilongjiangensis* was detected in *H. concinna* and *H. longicornis* but not in *D. silvarum* in a recent surveillance conducted in China (Liu et al., 2016), it remains unclear whether *D. silvarum* is a true host of *R. heilongjiangensis*. Therefore, additional isolates obtained in other geographic regions in Far East Asia, including other regions in Japan, need to be analyzed to fully understand the genomic diversity, phylogeography, and host association of *R. heilongjiangensis*.

### Detailed Genomic Comparison of *R. heilongjiangensis* and *R. japonica*

Duan et al. (2014) reported the results of a genomic comparison of *R. heilongjiangensis* strain 054 and *R. japonica* strain YH. However, the sequence of YH used for comparison has been found to contain significant numbers of sequencing errors (Akter et al., 2017). Therefore, we reanalyzed the genomic differences between the two closely related species using the genome sequences of the *R. heilongjiangensis* Sendai strain and the *R. japonica* YH_M strain (Akter et al., 2017).

The average nucleotide identity (ANIb) between the two strains was 99.2%. WGS alignment revealed perfect collinearity of the sequences and identified 7,359 SNPs and 157 InDels (>10 bp). The 157 InDels included two relatively large InDels (>1 kb). One was a 2.5-kb *R. heilongjiangensis*-specific region encoding a RelA/SpoT family protein, which was split into two CDSs and two hypothetical proteins. The other was a 1.1-kb region specific to *R. japonica* that corresponded to one of the sequences duplicated in *R. japonica*. A RelA/SpoT family protein is also encoded in this region and split into two CDSs (see below for a more detailed analysis of the genes encoding RelA/SpoT family proteins). The two regions existed in all sequenced strains in each species and were therefore species specific.

**Figure 2:**
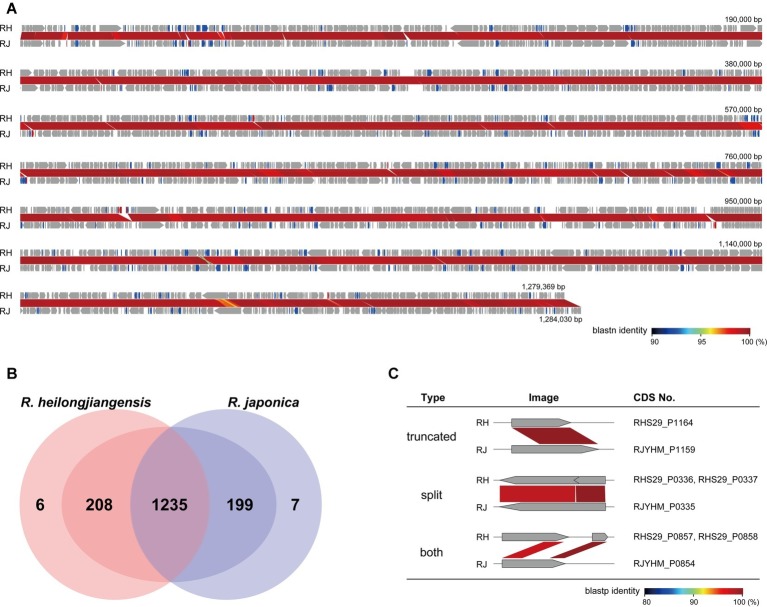
Detailed comparison of gene repertoires between *R. heilongjiangensis* and *R. japonica*. **(A)** Genome alignment and nucleotide sequence homology of *R. heilongjiangensis* Sendai-29 (RH) and *R. japonica* YH_M (RJ). Conserved, degraded and specific (absent or only very short homologous sequences were present in the other species) CDSs are indicated in gray, blue and red, respectively. This figure was constructed using GenomeMatcher. **(B)** Venn diagram showing the numbers of conserved or specific CDSs in *R. heilongjiangensis* and *R. japonica*. Only six and seven CDSs were truly specific to *R. heilongjiangensis* and *R. japonica*, respectively (see the main text for details). **(C)** Examples of degraded CDSs. Details of all CDSs are shown in Supplementary Table 2.

To compare the CDS repertoires, CDSs were identified using the same program (Prokka) and by manual inspection of all CDSs. Among the 1,449 and 1,441 CDSs identified in *R. heilongjiangensis* and *R. japonica*, respectively, 1,235 were clearly conserved between the two species (Figure 2; Supplementary Table 2). Although 214 and 206 CDSs were specific to *R. heilongjiangensis* and *R. japonica*, respectively, in the initial autoannotation, many of these CDSs were apparently degraded (split, truncated, or both) due to the presence of SNPs and small InDels (Figure 2; Supplementary Table 2). Only six CDSs were specific to *R. heilongjiangensis* (homologous sequences were absent or were only very short in *R. japonica*). Similarly, only seven CDSs were specific to *R. japonica*. The functional annotation of these CDSs indicated that most of these CDSs encode hypothetical proteins or apparently truncated proteins (Supplementary Table 2).

Analysis of the CDSs that have been degraded in either of the two species (Supplementary Table 2) revealed that most of these CDSs also encode small hypothetical proteins or proteins that appear to have already been truncated. Several of the CDSs may encode functional proteins, such as a phosphate acetyltransferase and a CorA-like magnesium transporter (both are split in *R. heilongjiangensis*). However, in the case of the phosphate acetyltransferase gene, the acetate kinase gene (located downstream of the phosphate acetyltransferase gene) was highly degraded in both species, indicating that the acetyl-CoA biosynthesis pathway has been inactivated in both species. In the case of the CorA-like magnesium transporter gene, another CDS encoding the magnesium transporter MgtE was conserved in both species, indicating that *R. heilongjiangensis* is also able to transport magnesium. Similarly, although a CDS encoding a putative 4alpha-hydroxy-tetrahydrobiopterin dehydratase appeared to be truncated only in *R. japonica*, both species and other SFG Rickettsia lack the tetrahydrobiopterin biosynthesis pathway because other components of the pathway are missing.

**Figure 3:**
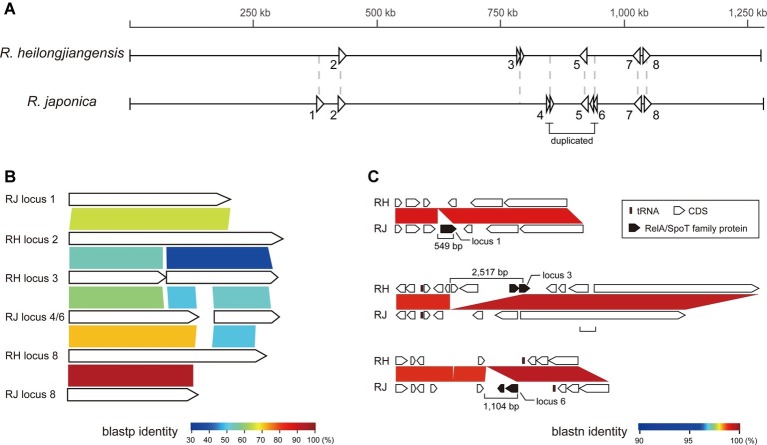
The genes encoding RelA/SpoT family proteins in *R. heilongjiangensis* and *R. japonica*. **(A)** The genomic locations of eight loci encoding RelA/SpoT family proteins in *R. heilongjiangensis* and *R. japonica*. **(B)** Blastp comparison of RelA/SpoT family proteins. The proteins encoded by loci 5 and 7 are not shown because they presented only low similarity to other proteins. **(C)** Three species-specific loci encoding RelA/SpoT family proteins.

The conservation of the genes encoding RelA/SpoT family proteins, which have been demonstrated in many bacteria to regulate (p)ppGpp production and contribute to the adaptation to environmental stress (Potrykus and Cashel, 2008; Atkinson et al., 2011), was complex. We identified eight loci containing RelA/SpoT family protein-encoding CDSs in either *R. heilongjiangensis* or *R. japonica* (Figure 3). Among these loci, three (loci 2, 5, and 7) were conserved in both genomes, and one (locus 8) was truncated in *R. japonica*. One (locus 3) and three (loci 1, 4, and 6) loci were specifically present in *R. heilongjiangensis* and *R. japonica*, respectively. Loci 3 and 6 correspond to the abovementioned 2.5-kb and 1.1-kb regions specific to *R. heilongjiangensis* and *R. japonica*, respectively. Although it has been reported that mutation of a RelA/SpoT-encoding gene affects the plaque phenotype in *R. rickettsii* (Clark et al., 2011), it is unknown whether or how the difference in the repertoires of RelA/SpoT family proteins between *R. heilongjiangensis* and *R. japonica* affects any phenotypes or resistance to environmental stress in the two species.

Taken together, the results of our detailed CDS comparison indicate that there is only a small difference in the gene repertoire between the two species. This finding implies that functional or physiological differences between the two species (at least between the two strains analyzed) may, if present, result from SNPs and small InDels rather than a difference in the gene repertoire. Importantly, however, the genomic regions found to be specific to each species, particularly the 2.5-kb and 1.1-kb regions specific to *R. heilongjiangensis* and *R. japonica*, respectively, can serve as ideal PCR targets for distinguishing the two species.

### Inter- and Intraspecies Variation in Tandem Repeats in the Genes Encoding Major Surface Proteins

As described above, we observed that tandem repeat sequences in the genes encoding major surface proteins were the major sources of InDels. A similar finding was obtained in a genomic comparison of tandem repeat structures in *R. japonica* (Akter et al., 2017). Therefore, we analyzed the inter- and intraspecies variation in tandem repeat structures in the *rompA*, *sca1,* and *sca2* genes using five *R. heilongjiangensis* and 32 *R. japonica* isolates to expand our previous analysis of 31 Japanese *R. japonica* isolates (Akter et al., 2017). The 32 *R. japonica* isolates included one Chinese isolate that was recently deposited in a public database (accession no. GCA_003454715.1, Supplementary Table 3). Note that only 12 *R. japonica* genomes were used for the analysis of *rompA* because the presence of tandem repeat sequences in the *rompA* genes of other isolates has not been determined.

**Figure 4:**
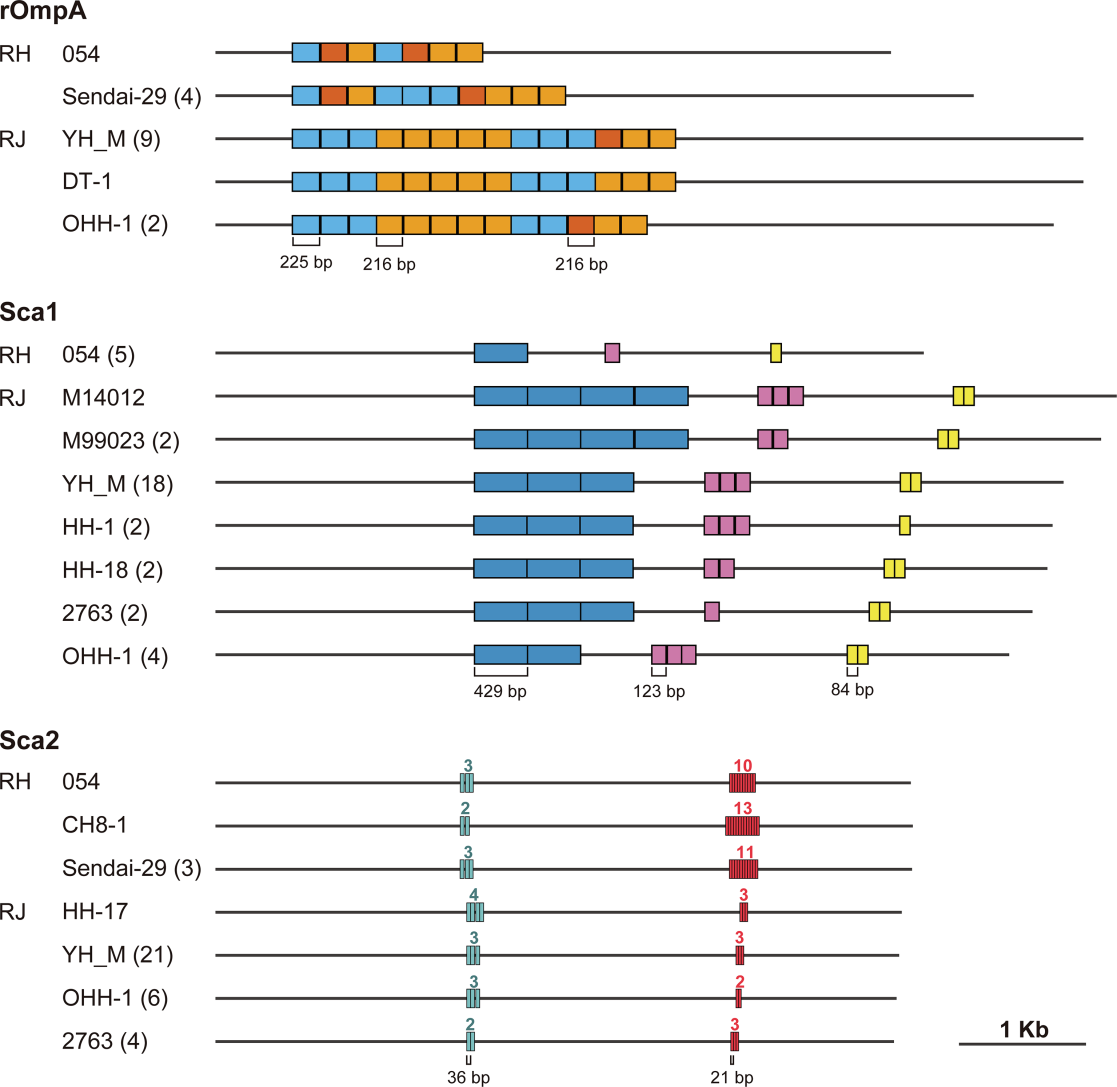
Tandem repeat structures in the *rompA*, *sca1*, and *sca2* genes of *R. heilongjiangensis* and *R. japonica* isolates. Tandem repeat sequences were identified using Tandem Repeats Finder. The names of the representative isolates exhibiting each structure are indicated with the number of isolates presenting the same structure in parenthesis. The numbers above each repeat region in the *sca2* gene represent the numbers of repeat units.

rOmpA has been associated with adherence to and invasion of human endothelial cells by interacting with integrin alpha2beta1 (Hillman et al., 2013). The tandem repeat structure was first analyzed in the *rompA* gene of *R. rickettsii* (Anderson et al., 1990), but the functions of the tandem repeats remain to be elucidated. Our analysis showed that the tandem repeat regions in *rompA* are composed of three very similar sequences of 225, 216, and 216 bp in the two species (Figure 4). Although a previous study of *R. rickettsii* (Anderson et al., 1990) defined two types of repeat units (type-I, 225 bp; type-II, 216 bp), we found that the type-II unit can be divided into two subtypes (type-IIa and type-IIb, see Supplementary Figure 1 for sequence alignment). While a certain level of intraspecies variation was detected in the two species, one striking interspecies difference was observed: the *R. japonica* genes contained more repeat units than the *R. heilongjiangensis* genes.

The Sca1 protein of *R. conorii* expressed in *Escherichia coli* has been demonstrated to mediate adhesion to mammalian epithelial cells (Riley et al., 2010). While three types of repeat sequences showing remarkable intraspecies variation were identified in the *sca1* genes of *R. japonica*, no tandem repeats were identified in the *R. heilongjiangensis* genes.

Functional and structural analysis of the Sca2 proteins of *R. conorii*, *R. rickettsii* and *R. parkeri* revealed that Sca2 functionally mimics the host protein formin and promotes the nucleation and elongation of actin filaments and the actin-based intracellular motility of *Rickettsia* cells (Haglund et al., 2010; Kleba et al., 2010; Cardwell, 2012; Madasu et al., 2013). Our analysis identified two types of tandem repeats in the *sca2* genes of *R. heilongjiangensis* and *R. japonica*. While both types of repeats showed intraspecies variation in each species, the 21-bp repeat has been markedly amplified in *R. heilongjiangensi*s.

The surface proteins encoded by these three genes have been demonstrated or are thought to be associated with various functions related to interaction with the host (Gillespie et al., 2015). The functions of the repeat structures in these proteins are currently unknown. However, as all tandem repeat-containing regions in the three proteins are exposed on cell surfaces, analyses of the inter- and intraspecies variation in these tandem repeats in a wider range of SFG *Rickettsia* species and assessment of the structure-function relationships of this variation may provide novel insights into the biology and pathogenicity of SFG Rickettsia.

## Conclusion

We determined the WGSs of four *R. heilongjiangensis* isolates, including three Japanese isolates and one Chinese isolate. The identical WGSs of the Japanese isolates indicate that *H. concinna* ticks carrying a single *R. heilongjiangensis* clone have been distributed in Sendai, Japan. Genomic comparison of the *R. heilongjiangensis* isolates including a previously sequenced Chinese strain revealed that, while some sign of geographic separation was noted, the *R. heilongjiangensis* isolates showed very low genomic diversity, comparable with that in their close relative *R. japonica*. We further performed a detailed comparison of the CDS repertoires between *R. heilongjiangensis* and *R. japonica* and accurately identified conserved and species-specific CDSs. The results of this analysis revealed that there is only a small difference in gene repertoires between the two species, suggesting that SNPs and small InDels are mainly responsible for the functional or physiological differences between the two species. Several species-specific genomic regions were also identified in this analysis, which can serve as ideal PCR targets for distinguishing *R. heilongjiangensis* and *R. japonica*.

## Data Availability Statement

The datasets generated for this study can be found in the DDBJ/EMBLE/NCBI under accession numbers AP019864 (strain Sendai-29), AP019865 (strain Sendai-58), AP019863 (strain HCN-13), AP019862 (strain CH8-1), and DRA008226 (Illumina raw data).

## Author Contributions

KK and TO performed sequencing analysis and genome assembly. HF, SY, and SA isolated *R. heilongjiangensis* and prepared genomic DNA. KK analyzed the data with support from YG, YO, SA, and TH. KK and TH wrote the manuscript. All authors gave final approval for the manuscript.

### Conflict of Interest

The authors declare that the research was conducted in the absence of any commercial or financial relationships that could be construed as a potential conflict of interest.

## Footnotes

^1^http://ftp.ncbi.nlm.nih.gov/genomes/refseq/

^2^http://platanus.bio.titech.ac.jp/pltanus_trim

^3^http://broadinstitute.github.io/picard/

^4^https://github.com/wdecoster/nanofilt
