# Supplementary material for: Genomic Features of Rickettsia heilongjiangensis Revealed by Intraspecies Comparison and Detailed Comparison With Rickettsia japonica
Source: Front Microbiol. 2019 Dec 6;10:2787. doi: 10.3389/fmicb.2019.02787 (PMC6908463; doi:10.3389/fmicb.2019.02787)
Supplement: Supplementary file 2 [file Image_1.pdf]

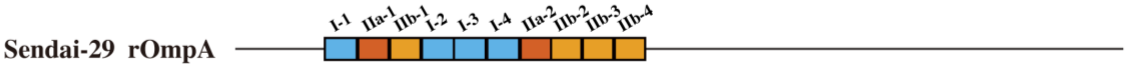


```
I-1     1>ATAGGTAATACAAATTCATTAGACACGATAAATGTAGGAGCAGGTACAGCCAC--GT--TAGGGGGAGCGGTTATTAAAG>76
I-2     1>ATAGGTAATACGGCAGCATTAGCAACAGTGAATGTAGGAGCAGGTACAGCCAC--GT--TAGGGGGAGCGGTTATTAAAG>76
I-3     1>ATAGGTAATACAAATTCATTAGACACGATAAATGTAGGAGCAGGTACAGCCAC--GT--TAGGGGGAGCGGTTATTAAAG>76
I-4     1>ATAGGTAATACAAATTCATTAGACACGATAAATGTAGGAGCAGGTACAGCCAC--GT--TAGGGGGAGCGGTTATTAAAG>76

IIa-1   1>ATAGGTAATACAAATTCATTAGACACGATAAATGTAGGAGCAGGTACAGCCAC--GT--TAGGGGGAGCGGTTATTAAAG>76
IIa-2   1>ATAGGTAATACAAATTCATTAGACACGATAAATGTAGGAGCAGGTACAGCCAC--GT--TAGGGGGAGCGGTTATTAAAG>76

IIb-1   1>ATAGGTAATACGGCAGCATTAGCAACAGTGAATGTAGGAGCAGGTTT-GCTACAAGTACAAGGCGGAGTGG-TA--AAAG>76
IIb-2   1>ATAGGTAATACGGCAGCATTAGCAACAGTGAATGTAGGAGCAGGTTT-GCTACAAGTACAAGGCGGAGTGG-TA--AAAG>76
IIb-3   1>ATAGGTAATACGGCAGCATTAGCAACAGTGAATGTAGGAGCAGGTTT-GCTACAAGTACAAGGCGGAGTGG-TA--AAAG>76
IIb-4   1>ATAGGTAATACGGCAGCATTAGCAACAGTGAATGTAGGAGCAGGTTT-GCTACAAGTACAAGGCGGAGTGG-TA--AAAG>76


I-1     77>CTACTACGACTAAA-TTAACAAATGCTGCGTCGGTATTAACCCTTACAAATGCAAATGCAGTATTAACAGGTGAGATTGA>155
I-2     77>CTACTACGACTAAA-TTAACAAATGCTGCGTCGGTATTAACCCTTACAAATGCAAATGCAGTATTAACAGGTGAGATTGA>155
I-3     77>CTACTACGACTAAA-TTAACAAATGCTGCGTCGGTATTAACCCTTACAAATGCAAATGCAGTATTAACAGGTGAGATTGA>155
I-4     77>CTACTACGACTAAA-TTAACAAATGCTGCGTCGGTATTAACCCTTACAAATGCAAATGCAGTATTAACAGGTGAGATTGA>155

IIa-1   77>CTACTACGACTAAA-TTAACAGATGCTGCGTCAGCAGTGACATTTACGAATCC---TGTAGTGGTAACCGGAGCGCTAGA>152
IIa-2   77>CTACTACGACTAAA-TTAACAGATGCTGCGTCAGCAGTGACATTTACGAATCC---TGTAGTGGTAACCGGAGCGCTAGA>152

IIb-1   77>CGAATGC-AATAAACTTAACGGATAATGCGTCAGCAGTGACATTTACGAATCC---TGTAGTGGTGACCGGAGCGCTAGA>152
IIb-2   77>CGAATGC-AATAAACTTAACGGATAATGCGTCAGCAGTGACATTTACGAATCC---TGTAGTGGTAACCGGAGCGCTAGA>152
IIb-3   77>CGAATGC-AATAAACTTAACGGATAATGCGTCAGCAGTGACATTTACGAATCC---TGTAGTGGTAACCGGAGCGCTAGA>152
IIb-4   77>CGAATGC-AATAAACTTAACGGATAATGCGTCAGCAGTGACATTTACGAATCC---TGTAGTGGTAACCGGAGCGCTAGA>152


I-1     156>TAACACCACAGGTGGTGATAATGTAGGTGTCTTAAATTTAAATGGTGCATTAAGTCAAGTAAGTGGGAAT>225
I-2     156>TAACACCACAGGTGGTGATAATGTAGGTGTCTTAAATTTAAATGGTGCATTAAGTCAAGTAAGTGGGAAT>225
I-3     156>TAACACCACAGGTGGTGATAATGTAGGTGTCTTAAATTTAAATGGTGCATTAAGTCAAGTAAGCGGGAAT>225
I-4     156>TAACACCACAGGTGGTGATAATGTAGGTGTCTTAAATTTAAATGGTGCATTAAGTCAAGTAAGTGGGAAT>225

IIa-1   153>TAATACC---GGTAATGCAAATAATGGTATAGCAACGTTTACCGGT-AAT--AGTACAGTAACTGGCAAT>216
IIa-2   153>TAATACC---GGTAATGCAAATAATGGTATAGCAACGTTTACCGGT-AAT--AGTACAGTAACTGGCAAT>216

IIb-1   153>TAATACC---GGTAATGCAAATAATGGTATAGCAACGTTTACCGGT-AAT--AGTACAGTAACTGGCAAT>216
IIb-2   153>TAATACC---GGTAATGCAAATAATGGTATAGCAACGTTTACCGGT-AAT--AGTACAGTAACTGGCAAT>216
IIb-3   153>TAATACC---GGTAATGCAAATAATGGTATAGCAACGTTTACCGGT-AAT--AGTACAGTAACTGGCAAT>216
IIb-4   153>TAATACC---GGTAATGCAAATAATGGTATAGCAACGTTTACCGGT-AAT--AGTACAGTAACTGGCAAT>216
```

Supplementary Figure 1. Sequence alignment of the repeat units in the *rompA* gene of *R. heilongjiangensis* strain Sendai-29.

The units are classified into type I, type II-a, and type II-b. Nucleotides that differ from those in type I-1 are highlighted in orange.
